# Power Dissipation of an Inductively Coupled Plasma Torch under E Mode Dominated Regime

**DOI:** 10.3390/mi12070834

**Published:** 2021-07-18

**Authors:** Nan Yu, Renaud Jourdain, Mustapha Gourma, Fangda Xu, Adam Bennett, Fengzhou Fang

**Affiliations:** 1 Centre of Micro/Nano Manufacturing Technology, University College Dublin, D04 V1W8 Dublin, Ireland; fengzhou.fang@ucd.ie; 2 Institute for Materials and Processes, University of Edinburgh, Edinburgh EH9 3FB, UK; 3 Surface Engineering and Precision Institute, Cranfield University, Cranfield MK43 0AL, UK; r.jourdain@cranfield.ac.uk (R.J.); gourma.m@gmail.com (M.G.); a.d.bennett@cranfield.ac.uk (A.B.); 4 Department of Mechanical Engineering, University of Bath, Bath BA2 7AY, UK; F.Xu@bath.ac.uk

**Keywords:** heat transfer, plasma processing, inductively coupled plasma

## Abstract

This paper focuses on the power dissipation of a plasma torch used for an optical surface fabrication process. The process utilizes an inductively coupled plasma (ICP) torch that is equipped with a De-Laval nozzle for the delivery of a highly collimated plasma jet. The plasma torch makes use of a self-igniting coil and an intermediate co-axial tube made of alumina. The torch has a distinctive thermal and electrical response compared to regular ICP torches. In this study, the results of the power dissipation investigation reveal the true efficiency of the torch and discern its electrical response. By systematically measuring the coolant parameters (temperature change and flow rate), the power dissipation is extrapolated. The radio frequency power supply is set to 800 W, E mode, throughout the research presented in this study. The analytical results of power dissipation, derived from the experiments, show that 15.4% and 33.3% are dissipated by the nozzle and coil coolant channels, respectively. The experiments also enable the determination of the thermal time constant of the plasma torch for the entire range of RF power.

## 1. Introduction

For the past two decades, researchers have investigated the design and the use of inductively coupled plasma (ICP) torches for scientific and industrial applications. During this period, ICP torches were proven to be an extraordinary tool for material scientists and surface engineers. Researchers are now broadening the capabilities of ICP torches to enable the correction of large ultra-precise optical components. The worldwide demand for ultra-precise surfaces led to the creation of a dwell time surface correction technique using plasma figuring [1]. The technique is based on the use of a unique ICP torch that is both presented and scrutinized from a thermal viewpoint. This work supports the scientific understanding of ICP torches and their processing capability. 

**Figure 1:**
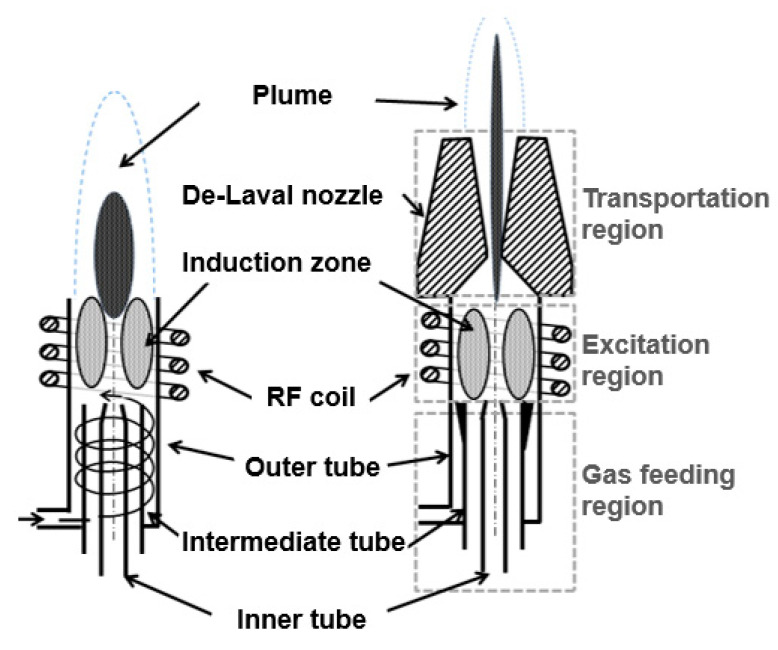
Regular ICP torch (**left**); ICP torch under investigation (**right**).

Industrial applications using plasma at atmospheric pressure include the surface modification [2] and etching [3,4,5] of different material surfaces. Plasma technologies actualize cost-effective processing conditions, compared to vacuum-based processes. This consideration is noteworthy for correction at the nanometer level of meter class optics because the processing of large optics requires vacuum chambers that are expensive and increase the lead time. The ICP torch (see Figure 1, right) under investigation is made of three distinctive sets of parts: nozzle, coil and a set of gas feeding tubes. The reactive gas is introduced in the core of the plasma using the inner tube. The 1 mm aperture tube enables the creation of a high number of free radicals that are transported along the main axis of symmetry. Consequently, the torch carries out the effective etching of most silicon-based materials. To investigate the aerodynamic characteristic of the plasma jet, the authors carried out a computational fluid dynamic (CFD) study in 2016 [6]. Thus, temperature, pressure and velocity distributions of the plasma jet are known, and its thermodynamics is understood. However, the power dissipation mechanism of this torch was not investigated at the time. 

The torch under investigation is better presented through its three well-defined regions: gas feeding, excitation and transportation. The gas feeding region provides a consistent and controlled flow rate of gas through the use of three coaxial tubes. Gas excitation (i.e., ionization and dissociation) takes place in the excitation region where the plasma core temperature is above 10,000 °C [7]. In this region, the radio frequency (RF) power, which is the transmission of the output power of a transmitter to an antenna, is transferred into the plasma by the helical coil. Finally, a large De-Laval nozzle is mounted onto the outlet of the ICP torch for securing a highly collimated plasma jet. In fact, this nozzle is used for tailoring the jet diameter without requiring additional mechanical or electrical modifications to the ICP torch. Notwithstanding, a large amount of RF power was contained within the small volume of the induction zone. This constraint led to a significant challenge in maintaining optimum performance over long process durations. Considering the developed ICP torch was typically used for 50 min when 400 mm side to side surfaces were processed and the theoretical duration for 1 m square optical surfaces was estimated to be 10 h [7], a more detailed understanding of the ICP torch was required. This deeper understanding was underpinned by the CFD study carried out previously [6], and by developing an understanding of the power dissipation mechanisms in the plasma figuring process.

Regular ICP torches (see Figure 1, left) are characterized by a less complex mechanical design. Indeed, the coil is wound around the outer tube. Additionally, the set of coaxial tubes is entirely made of fused silica. These tubes are often named quartz tubes. Regular ICP torches are well documented, and it is possible to understand their energy dissipation mechanisms. In fact, they are radiation, heat transfer to the walls and increase enthalpy to the gas. Reed published the first paper on regular ICP torches, where 55% of the RF power was transferred to the core area [8]. Boulos et al. worked on the mathematical and experimental analysis of ICP torches over the past decades [9]. Chen added a 2D electromagnetic (EM) field in the numerical model and studied the energy transfer of the plasma system [10]. Most recently, Lee [11] reviewed the fundamental understanding of hysteresis physics in ICP plasmas and their application. 

The RF plasma torches reported in the aforementioned publications are significantly different to the torch developed in this study for making like-for-like comparisons. Indeed, regular ICP torches do not have nozzles and the outer quartz tube tends to be short. Still, it seems reasonable to estimate that 50% of the RF power is potentially coupled with the core of the plasma. In addition, it is established that the power losses into the torches are largely driven by heat transfer to the walls. However, none of the publications have reported results about the temperature of the coolant that channels through the coil. The authors believe that the lack of information is because clear evidence exists of torch performance degradation due to poor coil surface integrity. Indeed, oxidation takes place when coil temperature increases. Broadly, the RF signal energy coupled with the core of the plasma led to numerous investigations using diagnostic tools and methods. 

Many studies that focused on the temperature of an ICP torch jet were conducted by direct measurements, using probes and optical emission spectra. For example, Rahmane et al. [12] highlighted that the performance of an enthalpy probe is comparable with that of optical emission spectroscopy (OES). Fiebrandt [13] presented the RF ICP discharges by means of a Langmuir probe and concluded that the RF distortion and probe tip contamination were two common problems. The plasma torch under investigation did not allow the use of diagnostic tools for the following two reasons. Firstly, the torch was equipped with a nozzle that had a long and narrow outlet. Secondly, during the processing conditions, the distance between the torch nozzle outlet and substrate surface—6 mm—did not enable the positioning of either probes or optical fiber. However, it was possible to observe that the torch under investigation had an unusual E-H mode transition (plasma status change between E mode and H mode), which is introduced in Section 2 in detail. 

The work in this study focused on the power dissipation investigation of the developed ICP torch operated under E mode (capacitively coupled method or electrostatic electron heating mode [14]) and at atmospheric pressure. The large range of RF power for which the torch remained in E mode highlighted irregular behavior. From an experimental viewpoint, temperature measurements were carried out in steady state. The coolant flow rate and the temperature measurements were carried out for both plasma torch coil and nozzle coolant channels. In addition, the flow rates of both gas and coolant were monitored and investigated. The results are scrutinized to assess the effects of these variations on the physical response of this unique ICP torch. 

## 2. Main Issues in ICP

### 2.1. The Plasma Modes of ICP Torches: E Mode and H Mode

**Figure 2:**
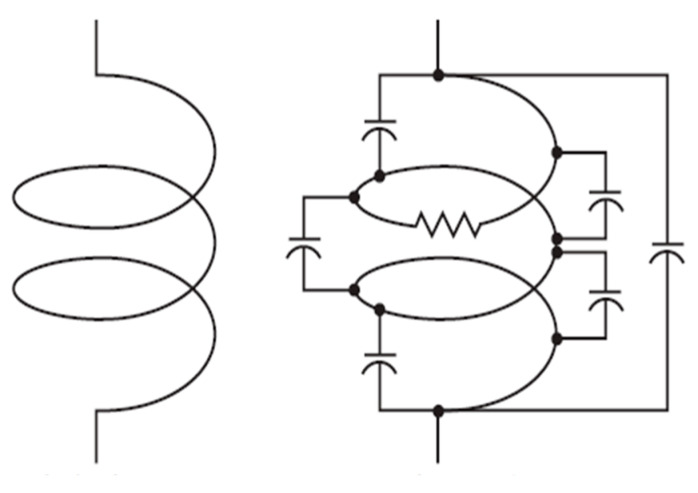
Coil: ideal inductor (**left**), inductor with stray capacitance and resistance (**right**).

For decades, plasma scientists have published work about the existence of various distinctive plasma modes. In the case of ICP torches run at atmospheric pressure, the plasma modes are dominated either by electric or by magnetic fields. They are named E mode (capacitively coupled) and H mode (inductively coupled, or EM electron heating mode). Figure 2 shows the ideal and real electrical schematic of the coil. 

The E mode plasma is characterized by both low electron density, high plasma potential, faint light emission and a short existence duration. Razzak et al. reported a 0.5 to 1 ms E-H mode transition, using a 4500 frame per second high-speed camera [15]. For regular ICP torches, the E mode plasma originates from a voltage drop across the exciter and the coil. On the other hand, the H mode plasma is characterized by the induced magnetic fields. 

The E-H mode transitions also exhibit hysteresis, i.e., when reducing input RF power starting in H mode, the transitions to E mode occur at lower powers than the transitions to H mode when starting in E mode. These phenomena were observed in the torch under investigation systematically. The developed torch energy coupling is mentioned hereafter. 

### 2.2. Energy Coupling during E-H Mode Transition

A basic understanding of the energy coupling between induction coil and RF discharges during ignition phase at atmospheric pressure range is provided hereafter. During E-H transition, plasma loading resistance increases dramatically, while the coil loading inductance decreases [15]. Razzak explained that during this transition, both frequency and phase shift from their resonance value, thereby deviating the output programmable logic controller (PLC) circuit, including the RF induction coil, from its ideal resonance frequency. Power coupling efficiency from the RF generator to plasma torch core area decreases due to the switching losses of the static inverter transistor elements. Later, Razzak et al. [16] utilized a constant-current high-power emittance conversion circuit to improve power efficiency. Further fundamental explanations about energy coupling can be found in [17]. It is evident that more should be revealed about the torch under investigation. 

### 2.3. The Unusual Long E Mode Range of the Plasma Torch

**Figure 3:**
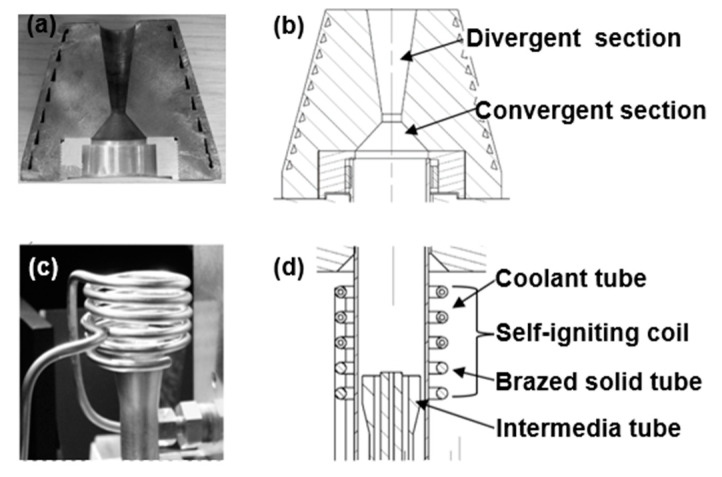
The ICP torch under investigation. (**a**) De-Laval nozzle photo; (**b**) CAD model: cross section of the nozzle; (**c**) photo of the self-igniting coil and intermediate tube; (**d**) CAD model: cross section of the torch.

In this work, the presence of both a nozzle, a self-igniting coil [18] and an intermediate tube made of conductive material (Figure 3) led to an unusually large RF power range for which diffused plasma was observed. Authors suggest that the plasma was under E mode for RF power in the range 200 W up to 800 W. In this study, the RF power refers in particular to the power delivered to the ICP torch, i.e., forward power from the RF generator minus reflected power from the RF network. The reason for the large E mode range was thought to be due to the voltage difference between the coil and the alumina intermediate tube. This explanation is different from the one given for regular ICP torches. The alumina tube was earthed and positioned a millimeter below the coil. In addition, it must be mentioned that a solid section was brazed onto the coil water cooled section and it largely surrounded the intermediate tube. 

For information, the E-H mode transitions were systematically accompanied by an abrupt change in the RF network impedance, which the free running RF signal generator compensated by adjusting the signal frequency within the 38 MHz to 42 MHz range. The E-H mode transition had occurred by 800 W. Consequently, the subsequent thermal investigation focuses on the E mode plasma operation, and will provide an alternative solution for the in-process measurement and monitoring of the power dissipation in the impinging plasma system.

## 3. Experimental Approaches

### 3.1. Plasma Delivery System

**Figure 4:**
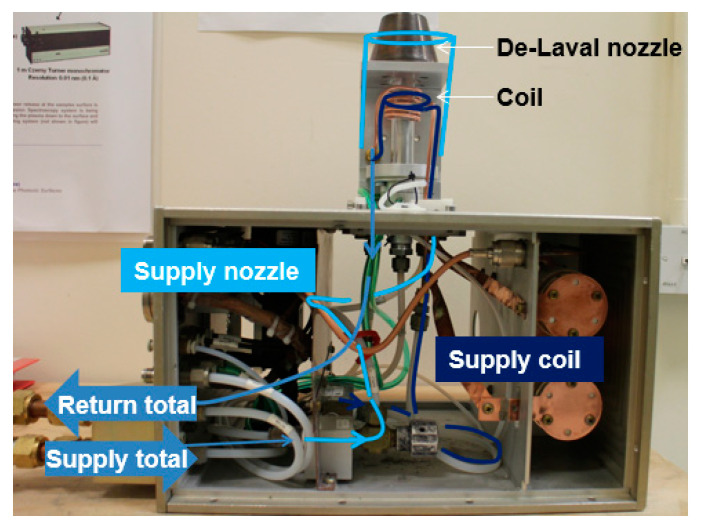
Torch assembly: plasma torch, coolant circuit, and fixed match RF network.

The torch assembly (see Figure 4) illustrates the part of the plasma delivery system used for all the measurements presented and analyzed in Section 4 and Section 5. The torch assembly is composed of the ICP torch, a fixed match RF network [19] and a coolant circuit. Coolant flows through the two main torch components: the coil—excitation region—and nozzle—transportation region. Coolant is highly important to protect the coil and copper nozzle from oxidation and melting. The nominal temperature of the coolant was 20 °C. 

**Figure 5:**
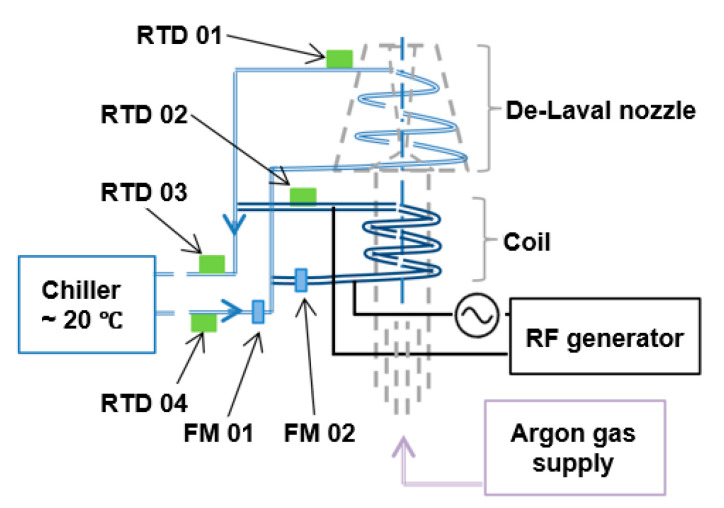
Plasma delivery system schematic.

Figure 5 illustrates the coolant distribution, the instrumentation of the torch assembly, the locations of flow meter, the locations of the temperature sensors, the chiller unit, the RF generator, the coil, the nozzle and the gas supply. In Figure 5, the acronym RTD and FM stands for resistance thermal device and flowmeter, respectively. Section 3.2 and Section 3.4 provide detailed information about both coolant flow rate and temperature measurements, respectively. 

### 3.2. Coolant Flow Rate Measurement

A Platon NGX series glass variable area flowmeter was used for measuring the flow rate of both torch coil and nozzle coolants. The accuracy of the flowmeter was ±1.25% full-scale deflection (±0.625 cm^3^/min). 

The flow rate of the torch coil coolant was set to 0.38 LPM (liter per minute) using a manually operated valve. Torch nozzle coolant flow rate was set to 1.18 LPM. The coolant was a mixture of de-ionized water and ethylene glycol. The 50/50 mixture by mass ethylene glycol has a specific heat capacity of three quarters of pure water (C_coolant_ = 3.14 kJ/(kg·K)). 

### 3.3. Coolant Supply

Coolant was provided to the plasma torch using a 19.4 KW cooling capacitance chiller (TAEevo Tech 051, MTA, Tribano, Italy). The distance between the chiller and the machine was 10 m. The total amount the coolant (tank and pipe) was 115 L. 

### 3.4. Temperature Measurement 

**Figure 6:**
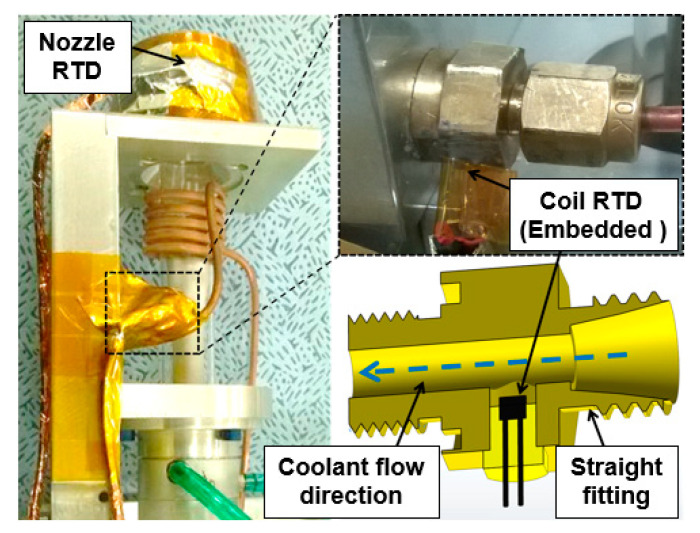
Instrumented plasma torch using RTD sensors.

Resistance thermal devices (RTDs) type PT100 class A (OMEGA Engineering, Manchester, UK) and a data acquisition (CompactDAQ) system (National Instruments, Austin, TX, USA) were used for temperature measurements and recording the data. All RTDs were calibrated in both icy water (0 °C) and boiling water (100 °C), respectively. The nonlinearity correction parameter determined was 0.385% per 100 °C. The RTDs were secured at key locations to measure the coolant temperatures of both inlet—supply total—outlet—return total—coil outlet and nozzle (Figure 5 and Figure 6 left). The instrumentation was carried out using a straight fitting mounted on the end of the coil (Figure 6, right). The RTD sensor was immerged in coolant. 

### 3.5. Gas Supply

Argon gas was supplied to the plasma torch using Brooks mass flow controllers (MFCs). Three MFCs were needed because the gas supply of the plasma torch is designed using three coaxial tubes. These MFCs were utilized to control and monitor the gas flow supplied when the RF power was ramped up from 250 W up to 800 W. In these experiments, the total nominal gas flow of argon was set to 22 LPM. 

**Figure 7:**
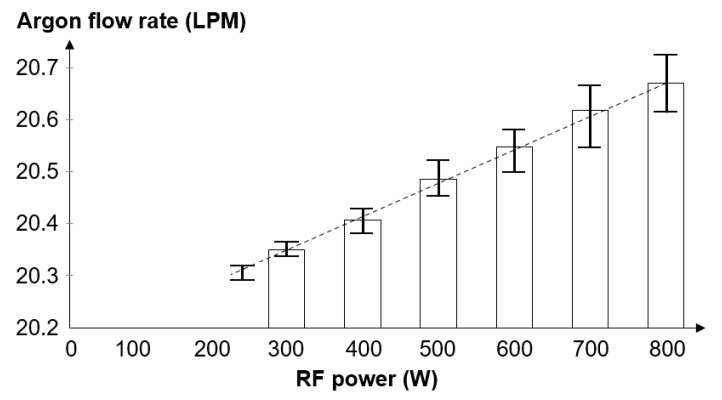
Argon flow rate versus RF power for the main gas flow.

The live value of the main gas flow was measured experimentally because a departure from nominal value was observed. These results (Figure 7) were obtained through a series of six experiments for which RF power was ramped up using 100 W steps. Recorded values showed an increase of 0.7 LPM for 800 W RF power. The mean deviation from the nominal gas flow was within 3.5%. 

### 3.6. RF Signal Generator 

**Figure 8:**
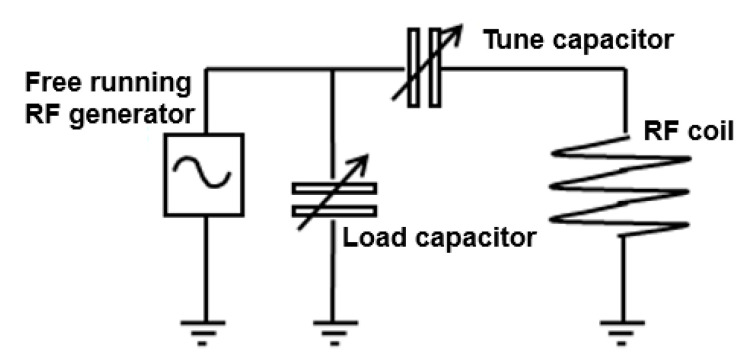
Highly simplified schematic of fixed match RF network.

RF power was provided by a Comdel CV 2000 (Comdel Inc., Gloucester, UK) that is a free-running signal generator. The ~40 MHz signal was provided to the plasma through a fixed match RF network (Figure 8). The signal generator had the capability to supply up to 2000 W of RF power. However, the experiments were carried out for a maximum power of 800 W because the plasma of the torch became instable above this value. The cause was the natural E-H mode transition of the plasma. In any case, the lack of control was thought to be due to the presence of the RTD sensors and wires located in the coil fitting (Figure 6). 

## 4. Analysis of the Power Dissipated in E Mode

### 4.1. Balance of Power Dissipation from the Plasma Delivery System

The RF power dissipation within the ICP torch (P_RF_) and the whole plasma delivery system is studied in this section, through deducing and calculating the power absorbed by coolant (P_coolant_) in two key components of this system (induction coil and De-Laval nozzle), as well as estimating the power consumed by argon gas (P_argon_) and plasma radiation (P_radiation_). Therefore, the dissipating rate of energy balance can be expressed as:

P_RF_ = P_coolant_ + P_argon_ + P_radiation_ (1)

In this study, experimental work was carried out to investigate the power dissipation of coolant part, while analytical calculations were conducted for argon gas and plasma radiation. 

### 4.2. Power Dissipated by Coolants

A series of temperature and flow measurements were carried out for the assessment of the power dissipated by the coolant. The main experimental parameters of the ICP torch are written in Table 1.

**Figure 9:**
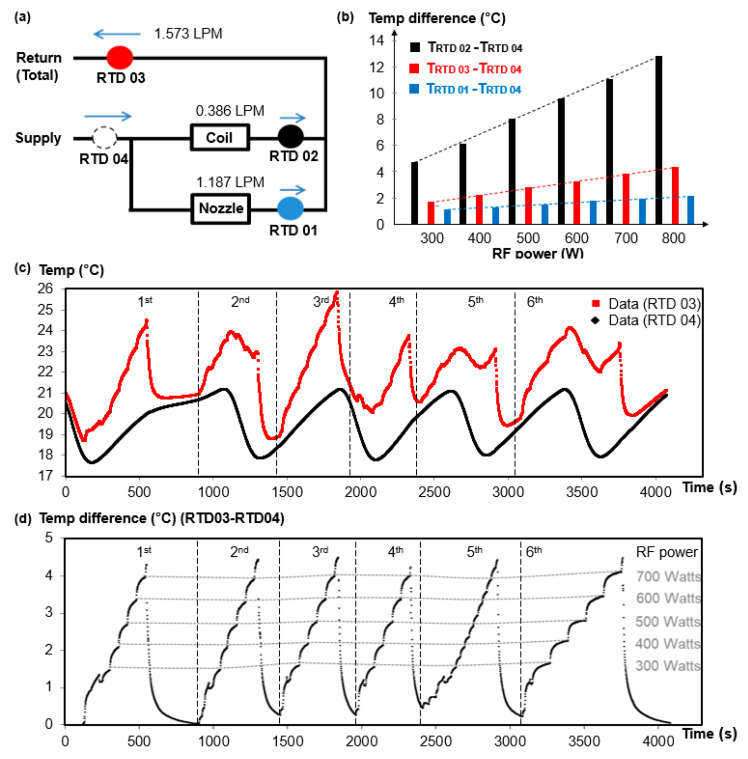
Schematic of coolant circuit and the temperature measurement data. (**a**) RTDs were positioned at four key points of the coolant circuit; (**b**) temperature difference of coolant channels: coil, nozzle and total; (**c**) original temperature reading logged from the RTD 03 and 04; (**d**) temperature difference between RTD 03 and 04.

During the experiments, the RF power was increased from 300.W up to 800 W. The RF power was stepped up by increments of 100 W that lasted 4 min. The test duration used for each increment enabled the torch components to reach thermal stability and consequently provided robust temperature records (Figure 9b). 

The temperature difference (TD) of the coolant between the inlet and coil or nozzle was calculated by the analysis of the recorded values obtained (Figure 9). The RTD positions in the coolant pipes are marked in Figure 9a, and a full procedure of the temperature measurement is demonstrated in Figure 9c: the temperature of RTD 03 (Return Total) was logged as the red line, and the temperature of RTD 04 (Supply) was logged as the black line. The supply coolant from the chiller is controlled between 18 °C and 21 °C. The measurement was carried out for six separate plasma ignition sequences, and in each case, the plasma was generated at around 250 W and the RF power was increased until E-H transition. The temperature difference between RTD 03 and RTD 04 is shown in Figure 9d, where a very stable TD value at each RF power is highlighted, with deviation of less than 0.2 °C. The temperature difference (TD) of the coolant between the inlet and coil or nozzle was calculated by the analysis of the recorded values obtained (Figure 9). The TD results for each RF power step are displayed Figure 9b. These results enabled one to correlate RF power and TD values for coolant channels at each key location where RTDs were set. 

These results were used to determine the power absorbed by the coolant. Therefore, for each RF power value the energy dissipation rate of the coolants was calculated using Equation (2).

**Figure 10:**
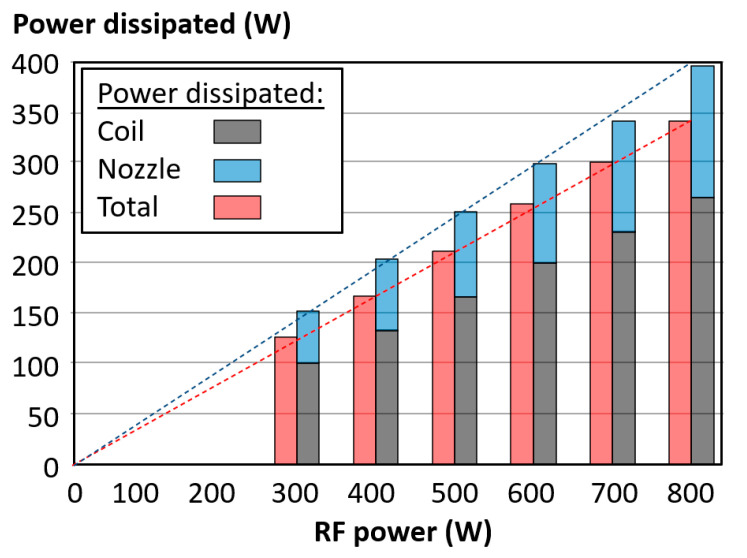
RF power versus power dissipated for each coolant channel.

ΔE_coolant_ = *C_pc_* · *v_c_* · ΔT (2)

where *C_pc_* = 3.14 kJ/(kg·K) is the specific heat capacity of coolant; *v*_c_ = 1055.7 kg/m^3^ × 1.558 L/min = 1.645 kg/min is the flow rate; ΔT is the TD value of coolants for each RF power value set. Substituting these experimental values into Equation (2), the power dissipated by the coolants was calculated and plotted in Figure 10. 

Figure 10 enables one to highlight that a near linear correlation exists between RF power values and both TD and power dissipated values for the range 300 W up to 800 W. The measured power dissipation from the sum of the coil and nozzle is higher than the measured power dissipation from the total channel, which is attributed to a constant amount of heat transferred through the coolant pipe. Two valuable formulas can be achieved from Figure 10: (1) power dissipation from coolant through both coil and nozzle, i.e., Power_coil+nozzle_ = 0.488 × RF power; (2) the power loss from the coolant pipe = Power_coil+nozzle_ − Power_total_ = 0.063 × RF power.

### 4.3. Uncertainty Analysis

The uncertainty analysis was carried out for the experimental work of the power dissipation analysis. Both temperature and mass flow rate measurements were scrutinized. The uncertainty was estimated according to the method described by Dietrich [20]. The uncertainty budget is displayed and detailed in Table 2. First, the standard uncertainty of repeated readings (SURR) called *a* was calculated.

(3) $$a=\frac{s}{\sqrt{n}}$$

where *s* is the standard uncertainty, and *n* is the number of measurements. 

Then, either the resolution uncertainty named *b* or the correction uncertainty named *c* were calculated (see paragraph below), dependent of the experimental setup. Finally, the combined uncertainty was calculated.

(4) $$\mathrm{Combined}\;\mathrm{uncertainty}=\sqrt{a^{2}+b^{2}+c^{2}+\ldots \mathrm{etc}.}$$

where *a*, *b*, *c* are the standard uncertainties of each item that are taken into account. The expended uncertainty calculation uses the coverage factor (k) equal to 2. 

The mean TD at 800 W RF power was 12.9 °C (Figure 9b), and the standard deviation was 0.2 °C. Consequently, the SURR value of the mean TD based on ten readings was 0.0633 °C (i.e., 0.2/√10). On the other hand, the resolution of reading provided by the system of acquisition was 10^−6^ °C. Thus, the expanded uncertainty was 126.6 × 10^−3^ °C. 

The mean mass flow rate of the total coolant of three repeated readings was 1.645 kg/min (i.e., 1.56 LPM). The estimated standard deviation was 20 g/min; then, the SURR value was 11.55 g/min (i.e., 20/√3). On the other hand, the accuracy of flow meter was ±1.25%. Then, the correction uncertainty was 20.56 g/min. Thus, the expanded uncertainty was 30.93 g/min. 

The uncertainty of the power dissipation for an RF power set at 800 W was estimated. The result of coolant power dissipation was 390 W and the uncertainty was 0.2 W. The investigation provided a confidence level of 95% on the uncertainty calculated. 

## 5. Discussion

The work emphasized the flow and temperature measurements of coolant in the two key components of the developed plasma torch. All the experiments were conducted in the E mode plasma and the RF power was set below 800 W. Several aspects that affected the measurements were taken into account and scrutinized. They were presented in Section 4.2 and Section 4.3. An estimated uncertainty value of 0.2 W was calculated based on a standard uncertainty method. This analysis provided a level of confidence of 95%.

### 5.1. Experimental Approach Analysis 

The experimental approach enabled one to derive the power dissipated by the coolant in key components of the plasma torch. The RTDs provided a means to record the coolant temperatures at specific locations. However, the presence of RTDs closed to the induction coil (i.e., antenna) led to some disturbances of the RF network. Indeed, it was observed that the free-running RF generator could not re-tune the frequency during and after the E-H mode transition. This issue led to reflected power rushes. However, the method was proven robust for RF powers up to 800 W. This was unlike the impedance around most RF plasma system designs, where 50 Ω was found to be a good compromise in the context of early coaxial cables. Here, in this project, the plasma system uses a fixed match network with details published in [19] to meet the requirement of large optical fabrication purpose; hence, system resistance was not measured in the normal matched conditions.

It was highlighted that the less specific approach of temperature measurements that utilized the total supply and total return led one to underestimate the power dissipated by the coolant. Indeed, the sum of power dissipated by coil and nozzle coolant was 12.5% higher than the measured total power dissipated value (Figure 10). The authors suggest that the difference is due to the heat loss from the PTFA pipe between RTD 01/02 and RTD 03, as shown in Figure 5. The results show that the simplified TD measurement method is valuable for online assessments of the torch performance.

A 3.5% deviation from the nominal gas flow rate value was correlated with the maximum RF power. Unfortunately, the authors are not in the capacity to provide a firm explanation for this observation. In any case, the uncertainty was only 0.1%. 

### 5.2. Power Dissipation Analysis

The authors calculated the value of the total power dissipated by the coolant channels for an 800 W RF power. The value was 390 W (see Section 4.2, Figure 10). The investigation highlighted that the power dissipated in the nozzle coolant was 124 W. On the other hand, the power dissipated by the coil coolant was 266 W. 

The authors emphasize that power dissipation in the coil’s coolant is the sum of both radiations from the core of the plasma and ohmic losses in the coil surface due to the signal frequency. One knows that inductive currents flow through both the coil and matching network elements. Since the currents are large, ohmic losses in the circuit are significant. These losses at high frequencies are also higher than at low frequencies since the RF currents are constrained to flow near the surface of conductors. For copper, the depth of penetration also named skin depth δ is

δ = (2/(μ ω σ) ^1/2^) (5)

where μ is the conductor permeability ~0.999991, ω is the frequency ~40 MHz and σ is the conductivity 1.678 μOhm/cm. It is only about 10 μm. 

On the other hand, authors are aware that the power dissipated by the coil is larger than one reported by Jorabchi [18]. Based on that observation, one could think that the authors’ torch design affected the Q factor of the coil. Very likely, the presence of the alumina trumpet may have an effect. 

### 5.3. Power Dissipation and Torch Efficiency of the ICP Torch 

For regular ICP torches, the efficiency *ŋ* is the ratio of the plasma jet power *P*_plasma_ to the input RF signal power *P*_input_.

*ŋ* = *P*_plasma_/*P*_input_ (6)

For the authors’ ICP torch, a pseudo torch efficiency called *ŋ_p_* was created and calculated. The *ŋ_p_* value enables to calculate the amount of energy that has not been dissipated by the coolant. The *ŋ_p_* value is one minus the ratio of the coolant dissipated power to the input RF signal power.

*ŋ_p_* = 1 − (*P*_coolant_/*P*_input_) (7)

The value for *ŋ_p_* was 51.3% for an 800 W input RF power. The authors know that efficiency and pseudo efficiency were calculated in H and E mode, respectively, which further affect the relative value of *ŋ_p_*. In any case, the pseudo efficiency is discussed in the light of the regular ICP torch efficiency values. To do so, Table 3 displays the set of key values published in world leading research works. 

Table 3 highlights that less than 51.3% of the RF power was potentially transferred to the plasma bullet of the torch, and consequently, its jet. Additionally, the authors suggest that a fraction of the RF power was dissipated by the torch earth intermediate tube. There are two facts that support this claim. Firstly, the temperature difference between the plasma bullet and the intermediate tube is large. Indeed, as the intermediate tube does not melt, it can be assumed that its temperature is well below 600 °C. It should be highlighted that the intermediate tube is cooled down by convection due to the 22 LPM gas flow of argon that flushed the space between the outer and the inner tube [22]. Secondly, the electromagnetic (EM) fields are partially absorbed by the alumina due to its permeability constant. 

On the other hand, the result is given for the torch under investigation operated in E mode. For the time being, in H mode, the strong EM field affects the RTD sensors embedded in the coil and nozzle channels, so the power dissipation in H mode cannot be measured directly [23]. The authors are fully aware that in H mode, a better energy coupling takes place. In temperature measurement terms, the temperature difference of the coolant is likely to decrease because the EM fields are theoretically better coupled with the core of the plasma. For this research, the focus is to present an alternative method for in-process monitoring of the torch efficiency, typically for the plasma surface applications, e.g., plasma torch etching [24], surface treatment [25] and coating [26,27].

## 6. Conclusions

This study explores power dissipation during E mode ICP plasma generation. Temperature measurement was undertaken in the extreme environment of the plasma delivery system. The temperature increases of the coolant channels in both the coil and De-Laval nozzle were precisely measured. Consequently, the torch and its associated plasma delivery system are efficient and enable the discharge of a collimated plasma jet at atmospheric pressure. The main conclusions are drawn as below:

(1) The result showed for the very first time the temperature increases in the coil of an ICP torch. A total of 48.7% of the RF power was absorbed by the coolant in this ICP torch. This study highlighted the near linear correlation between the RF power and the dissipated power through the coolant channels of the two key components.
(2) The unique mechanical design of the developed torch experienced an E-H mode transition at circa 800 W RF power. Both the high RF power for E-H transition and the complex torch construction highlight the need for a robust RF generator and transmission line, in order to control the reflected power under E-H transition.
(3) The creation of the power dissipation assessment method can be implemented to monitor and assess the torch performance during the digitally supported plasma processing in the future.

## Figures and Tables

**Table 1:** Experimental parameters for the plasma torch.

Parameters	Values
Processing gas	Argon (research grade)
Gas flow rate	22 LPM
Radio frequency	~ 40.250 MHz
RF power	300, 400, 500, 600, 700, 800 W

**Table 2:** Uncertainty budget of the power dissipation of coolant.

Source of Uncertainty	Value (±)	Probability Distribution	Divisor	Uncertainty Value
**Temperature Measurement**	**10^−3^ °C**			**10^−3^ °C**
SURR	63.3	Normal	1	63.3
Resolution	10^−3^	Normal	2	5 × 10^−4^
Combined		Normal		63.3
Expanded		Normal (k = 2)		126.6
**Mass Flow Rate** **Measurement**	**g/min**			**g/min**
SURR	11.55	Normal	1	11.55
Operator reading	0.527	Normal	2	0.263
Instrument	20.56	Normal	2	10.28
Combined		Normal		15.46
Expanded		Normal (k = 2)		30.93

**Table 3:** Torch efficiency in literature (atmospheric pressure, gas: argon).

Researcher	Frequency (MHz)	Power (KW)	Flow Rate (LPM)	Regular ICP	Nozzle ICP	Plasma Mode	*ŋ* (%)	*ŋ_p_* (%)
Reed [8]	4	1.55	18.9	√		H	52–57	
Merkhouf [21]	4–5	25	100		√	H	55–60	
Chen [10]	3	1	20		√	H	68	
Authors’ torch	40	0.8	20		√	E		51.3
